# Wait Up!: Attachment and Sovereign Power

**DOI:** 10.1007/s10767-014-9192-9

**Published:** 2014-12-09

**Authors:** Robbie Duschinsky, Monica Greco, Judith Solomon

**Affiliations:** Social Work and Communities, Northumbria University, Newcastle City Campus, Ellison Pl, Newcastle upon Tyne, Tyne and Wear NE1 8ST, UK; Goldsmiths, University of London, Lewisham Way, New Cross, London SE14 6NW, UK; Center for the Family in Transition, 5725 Paradise Drive Bldg. B, Suite 300, Corte Madera, CA 94925, USA

**Keywords:** Attachment theory, Sovereignty, Politics, Lauren Berlant, Childhood

## Abstract

Sociologists and feminist scholars have, over many decades, characterised attachment as a social construction that functions to support political and gender conservatism. We accept that attachment theory has seen use to these ends and consider recent deployments of attachment theory as justification for a minimal State within conservative political discourse in the UK since 2009. However, we contest that attachment is reducible to its discursive construction. We consider Judith Butler’s depiction of the infant attached to an abusive caregiver as a foundation and parallel to the position of the adult citizen subjected to punitive cultural norms and political institutions. We develop and qualify Butler’s account, drawing on the insights offered by the work of Lauren Berlant. We also return to Foucault’s *Psychiatric Power* lectures, in which familial relations are situated as an island of sovereign power within the sea of modern disciplinary institutions. These reflections help advance analysis of three important issues: the social and political implications of attachment research; the relationship between disciplinary and sovereign power in the affective dynamic of subjection; and the political and ethical status of professional activity within the psy disciplines.

## Introduction

Attachment has become an important concept in queer theory, particularly in the work of Judith Butler and in discussions of her work (e.g. Ahmed 2001) and in the writings of Lauren Berlant. These scholars have developed the language of ‘attachment’ as a means of addressing the way that we emerge, within biographical time, thrown into a constellation of affections—which may have the quality of feeling necessary to our lives, but which may be both contingent and punitive. In the perspective offered by Butler, for example, it is accepted that children form an ‘attachment’ to their caregiver, and this attachment is considered as a significant process in itself for making sense of the political formation of subjects. The infant depends on the caregiver for protection and for the mastication of experiences and affects, and has a disposition to seek them when alarmed; as such, the attachment which forms for the caregiver means that each human subject emerges with an investment already present even for caregivers who are cruel or frightening. Butler offers the image of the alarmed infant looking for protection from such a caregiver as the condition and mirror of the adult political subject who emerges practically and affectively beholden to a normalising and potentially punitive culture and political institutions.

Such usage of the concept of ‘attachment’ occurs on the horizon of a parallel discursive practice within developmental psychology, ‘attachment theory’, which since the work of John Bowlby has been likewise founded on the observation that human infants, when alarmed, will seek comfort and protection from even an insensitive familiar caregiver and that the response of the caregiver to the infant’s alarm shapes their sense of self and the world in a significant, though not indelible, way. This psychological usage of the concept of ‘attachment’ has meant that from Oakley (1971) to Levine (2014) discourses on infant ‘attachment’ have been characterised as primarily ‘cultural ideology’ rather than having any meaningful referent. Contratto (2002; 29, 34), for instance, has characterised discourses on infant attachment since Bowlby as ‘profoundly conservative’, bent upon producing ‘familiar mother-blaming scenarios.’ We agree with critics of attachment theory that discourses invoking ‘attachment’ have often been deployed for normalising ends which, whilst claiming scientific credibility, find little or no justification in the available research evidence. We will show that this perspective can be well attested by considering the role of attachment within British political and policy discourse since 2009. However, we are excited by the way that scholars such as Butler and Berlant have considered the political implications of the scene of attachment itself, rather than considering attachment solely in terms of its discursive construction. We are interested to sustain a conversation between critical social theory and empirical research in child psychology regarding the role of interruption and intermission the affects which organise relational subjectivities. Our hope is that, for both fields, such a conversation can present opportunities for new questions and resources for thought.

Butler considers attachment through the lens of Foucault’s notion of ‘disciplinary power’, which pins an individual to their identity and sets up pressures and punishments so that they will regulate and normalise themselves. She is guided by his remarks in *Discipline and Punish*, though expresses concern that Foucault offers no adequate account of the affects of family life and that ‘the term “attachment” does not receive an independent critical analysis’ as a tool for social theory (Butler 2004a: 190). She urges the need for such work to discern the role of attachment in yoking us to disciplinary institutions and punitive cultural forms. Our argument will be that the attachment-subjugation relationship is less direct than Butler suggests. In developing this position, we take encouragement from Berlant and Edelman, who both make critical use of the concept and recommend further consideration: in *Sex, or the Unbearable* (2014), Edelman asks ‘What politics proceeds from undoing, from breakdowns in the subject’s forms of attachment?’, and Berlant urges the need ‘to make new terms for the encounter that restages the desire for attachment alongside all the ways that we fight it’ (Berlant and Edelman 2014: 66, 112). Our article will attempt a reconciliation and integration of empirical attachment research, constructionist critiques of attachment discourse and queer theorizations of the politics of the scene of attachment. In this, we attend to Berlant’s account in *Cruel Optimism* (Berlant 2011) of attachment as a scene through which children organise their optimism for living, and which provides opportunities for continuities and disruptions in the emergent terms of our enthrallment to our families, one another as adults, and to cultural and political forms. Attachment provides an important site at which, as Berlant explains, we not only receive subjugation as a particular kind of person (‘hey you!’) but also can address and change (‘wait up!’) our capacity to be affected with and by cultural forms and other people. We will deploy this account in considering the social and political implications of empirical attachment research; the relationship between discipline and sovereignty in the affective process of subjection and the political and ethical status of professional activity within the psy disciplines in interventions with families.

## Attachment Theory

‘Attachment’ was the English word used by the Stracheys in translating Freud’s genitive *Anlehnungs*, deployed in the *Three Essays on Sexuality* to refer to a kind of love which emerged on the back of (literally, ‘leaning-on’) the need of the infant for his or her caregiver for their self-preservation. Among the Anglophone psychoanalysts to pick up the term as a substantive, Bowlby (1958) made use of it in describing a disposition which promotes proximity-seeking behaviour in infants when they are hurt, alarmed or separated from their familiar caregiver. When activated, he called this disposition the ‘attachment system’ and anticipated that it would coordinate signals and movements including crying, smiling and crawling in attempts to gain proximity, and thus protection and emotional support, from the caregiver. For (Bowlby 1958: 167), attachment behaviour presumes upon a complementary response in the action of a ‘caregiver behaviour system’ which primes the attachment figure to retrieve the distressed infant. The mesh between attachment and caregiver retrieval systems thus functions to keep an attachment figure near and attentive to the child’s needs: ‘it is fortunate for their survival that babies are so designed by Nature that they beguile and enslave mothers.’ In line with other Object Relations theorists, Bowlby considered that the attachment relationship would help the child make meaning of the social world and of their own perceptual and affective responses to it. However, influenced by ethology, Bowlby (1969) also believed that the tendency for primate infants to develop attachments to familiar caregivers was the result of evolutionary pressures, since attachment behaviour would facilitate the infant’s survival in the face of dangers such as predation, exposure to the elements or attacks from conspecifics. Given this importance, Bowlby placed among the central tenants of attachment theory the idea that an attachment to at least one familiar caregiver would develop regardless of the sensitivity of this response from the familiar caregiver, so long as there is one available. Where the response from the caregiver to the infant’s alarm is sensitive, then the infant will anticipate that their caregiver will be responsive when they are distressed and not feel anxious in seeking out protection or support. However, Bowlby suggested that the infant’s disposition to seek the familiar caregiver when experiencing distress will be activated even when this caregiver is unreliable or rebuffs the child’s proximity-seeking behaviours.

In 1965, Ainsworth and Wittig designed the Strange Situation Procedure as a way of assessing individual differences in attachment behaviour. The Strange Situation Procedure is a laboratory-based procedure, divided into eight episodes. In the first episode, the infant and their caregiver enter a room with chairs situated a little away from a lovely heap of toys. The caregiver is asked to sit in one of the chairs. After 1 min, a person unknown to the infant enters the room and slowly tries to make acquaintance. The caregiver leaves the child with the stranger for 3 min and then returns. The caregiver departs for a second time, leaving the child alone for 3 min; it is then the stranger who enters and offers to comfort the infant. Finally, the caregiver returns and is instructed to pick the child up (Ainsworth and Wittig 1969). Around two thirds of infants in middle-class, non-clinical samples were found to use their caregiver as a ‘safe base’ from which to venture away to the toys. They showed distress and sought proximity with their caregiver on reunion, but could be comforted quite quickly, allowing them to return to play. This behaviour, termed ‘secure’ (B), suggests the activation of attachment behaviour on separation, and its subsidence once the infant feels confident that protection from their attachment figure is available. In line with Bowlby’s prediction, Ainsworth’s home observations, as well as subsequent research, found that the caregivers for such children were those most sensitive and responsive to the child’s attachment behaviour (e.g. Leerkes 2011).

However, a minority of infants in Ainsworth’s middle-class sample showed little visible distress on separation or reunion with their caregiver. This appeared to contradict Bowlby’s theory. However, Ainsworth theorised that the apparently unruffled behaviour of the avoidant infants is in fact a mask for distress—a hypothesis later evidenced through studies of the heart rate of avoidant infants (Sroufe and Waters 1977). Ainsworth, therefore, termed these infants ‘insecure-avoidant’ (A); this pattern was found in around a fifth of infants in middle-class and non-clinical samples. Her home observations found that the caregivers of these infants tended to rebuff attachment behaviour, which meant that it would be counterproductive for the infant to display distress or seek proximity. Ainsworth concluded that, when these infants experienced alarm and distress, they had learned that they had best not to communicate this. A third pattern was termed ‘insecure-resistant/ambivalent’ (C), and these infants showed distress even before separation and were frustrated and difficult to comfort on the caregiver’s return, seeming to distrust his or her availability even when the caregiver is present. In contrast to infants classified as ‘secure’ or ‘avoidant’, Ainsworth’s home observations revealed that the caregivers of the infants classified as ‘resistant/ambivalent’ were not reliable in their response to attachment signals. This third attachment pattern was found in around a tenth of infants in middle-class and non-clinical samples. Ainsworth concluded that these infants, when experiencing alarm and distress, were taking control of an otherwise unpredictable relationship by broadcasting their feelings and ignoring contingencies in their caregiver’s behaviour.

In the following years, the distinction between the three classifications was found to have high inter-rater reliability; the overwhelming majority of infants assessed at 11 or 12 months were placed in the same category 6 months later; the proportion of infants allocated to each of the categories was found to be equivalent across middle-class and non-clinical Western samples, as well as in countries such as Mali, Israel and Japan; and classifications from the Strange Situation Procedure were found to have a good predictive value for later child outcomes such as self-esteem and academic success (Solomon and George 2008). Yet a growing number of cases were found, from Ainsworth on, which did not fit the three patterns, and this was the case particularly in clinical and poverty samples (e.g. Egeland & Sroufe 1981; Crittenden 1983). Building on Bowlby and Ainsworth, a fourth attachment classification was added by Main and Solomon (1986, 1990), ‘disorganised/disoriented’ (D). This classification is not used alone, but is added to an A, B or C classification to identify the presence of a contradiction or disturbance in the sequencing of the infant’s behaviour with the caregiver in the Strange Situation, in particular during reunion behaviour. Infant behaviours coded as disorganised/disoriented include, for example freezing for more than 15 seconds on reunion with the caregiver with arms held at an awkward angle; crying angrily on reunion whilst turning in circles repeatedly away from, then towards, then away from the caregiver and striking the head or face on seeing the caregiver again. Like the other classifications, ‘disorganised/disoriented attachment’ with one caregiver little predicts the classification with another caregiver, which implies that the classification is tapping a quality of the relationship and not merely the child’s temperament (van IJzendoorn et al. 1999). Infant attachment classifications have been suggested by prospective longitudinal research to have an independent predictive value for later mental health. In the Minnesota Longitudinal Study, ‘in regression analyses, avoidant attachment scores, attachment disorganization ratings, elementary school behavior problem scores, and ratings of parent-child relationship quality at 13 years each contributed significantly to the prediction of psychopathology in adolescence‘ (Carlson 1998: 1120).

The ‘disorganised/disoriented’ classification has often been reified and misapplied as a merely a residual category for behaviour discrepant with the Ainsworth classifications (Duschinsky 2015). Where such a characterisation has occurred, it has made disorganisation/disorientation appear as the exhaustive addition to a four-part taxonomy, and attachment behaviour as having no logic besides the expression of free-standing taxonomic categories. Such a perspective misses the dynamic and patterned interplay of biological, social and political forces which generate the regularities which the classifications work to pick out (Duschinsky et al. 2015). A further point, often missed in reifications of disorganised/disoriented attachment, is that all infants experience countervailing affects. The issue of ‘what happens to the energy of attachment when it has no designated place?’ (Berlant 1998: 285) is not one only raised in the exception but, as Berlant documents, by degrees both raised and moderated within the dislocation and touch of everyday human experience; this is all the more true for infant experience where designation and place are still finding their feet. Disorganised/disoriented attachment is conceptualised as a limit case of this phenomenon, in which the attachment which directs an infant to seek the caregiver when alarmed is simultaneously countermanded and/or melted by some force issuing from the experience of the caregiving relationship itself in such a way that the infant is unable to coordinate a smooth behavioural response to reunion. As such, it is coded when an observer believes that the interrupted or disrupted quality of an infant’s behaviour in the strange situation suggests that optimism regarding finding in the caregiver a source of comfort and protection is being disrupted and dysregulated by feelings evoked in relation to the caregiver themselves, for example by the infant’s memories of the caregiver themselves as occasion for alarm, panic or despair. The ‘disorganised/disoriented’ (D) classification has been applied to around 12 % of infants in middle-class and non-clinical samples. Yet, since disorganised/disoriented behaviour is identified in upwards of 32 % of infants known to be neglected (Valenzuela 1990), and 48 % of infants known to be experiencing physical maltreatment from their caregiver (van IJzendoorn et al. 1999), this attachment classification has attracted attention from clinical and social welfare practitioners (Howe 2010; Shemmings & Shemmings 2014).

Particularly since the introduction of the ‘disorganised/disoriented’ attachment classification, assessments of infant attachment have been used in research on the role of cumulative risks in hindering caregiving. Socioeconomic risks are associated with higher rates of insecure attachment in general and of disorganised attachment in particular. A meta-analysis by van IJzendoorn et al. (1999) found that 25 % of infants from families in poverty were classified as showing ‘disorganised/disoriented’ behaviour in the strange situation, though subsequent studies have tended more towards 30 % (e.g. Fish 2001). Researchers found a cumulative effect of the social and economic difficulties faced by a family in predicting insecure attachment in infants (e.g. Shaw and Vondra 1993; Stansfeld et al. 2008). Broussard (1995), for example, found that 76.3 % of infants of adolescent mothers in poverty were classified as insecurely attached, and that by 14 months 63.6 % of the sample of infants displayed behaviour classified as disorganised/disoriented in the Strange Situation Procedure. Yet, the link between economic risk and infant attachment does not appear to be a direct one: Statistical analyses have found no direct relationship between the socioeconomic status of the family and the patterns of attachment shown by infants of that family. Instead, Raikes and Thompson (2005) used structural equation modelling to suggest that socioeconomic resources impact on attachment through two linked but distinct processes: First, by disturbing the capacity for emotion-regulation and emotional warmth by the infant’s caregiver(s); and second, by increasing chaos, conflict and distress in the general emotional climate of the home. Evidence that attachment is not reducible to socioeconomic risk, but has a developmental interaction with it, is that for children growing up in poverty a secure attachment with at least one caregiver has been found to markedly increase a child’s later well-being on a variety of measures (e.g. Sroufe et al. 1990).

With reference to disorganised/disoriented attachment specifically, a meta-analysis by Cyr et al. (2010) found that infants in non-maltreating families subject to five social and economic difficulties were just as likely to show disorganised/disoriented attachment behaviour in the Strange Situation as infants who were known to social services as having experienced physical abuse or neglect. The factors addressed by Cyr et al. included poverty, parental substance use, ethnic minority status within a country and a parent in adolescence. The researchers found that any of these factors alone had little association with a disorganised/disoriented infant attachment classification in the Strange Situation; it was their ramification which appeared to be predictive of the classification. Cyr et al. explain their findings through appeal to Solomon and George (1999), who theorise that the capacity of the caregiver to regulate themselves and their infant is undermined by the experience of chaos and helplessness which may be expected to result from the interaction of multiple social and economic problems. Furthermore, a meta-analysis conducted by Lyons-Ruth (1996: 68) concluded that social and economic risks may differentiate forms of disorganised attachment. She found that the infants ‘disorganized infant behavior in low-risk, middle-SES samples has been predominantly of the forced-secure subtype, in which the infant seeks contact with the caregiver without marked avoidance or ambivalence and is soothed by her presence but shows other unusual signs of hesitation, confusion, apprehension, dysphoria or conflict in relation to her.’ By contrast, ‘disorganized infant behavior in samples with serious social risk characteristics has been predominantly of the forced-avoidant subtype, in which prominent avoidant behaviour occurs in unexpected combination with distress, contact seeking, resistance, or other apprehensive or conflict behaviors.’ Lyons-Ruth’s longitudinal research has shown that these two groups of infants have distinct outcomes in later life—the former, for instance, showing more suicidal tendencies than the latter (Lyons-Ruth et al. 2013).

## Attachment and Political Conservatism

In sociological and feminist analyses, the idea of ‘attachment’ is treated as a discursive construction. Scholars observe how the appeal to biological instinct and the determinist findings of research are deployed to legitimise normative concepts of motherhood and to mandate social services and clinical inventions to police working class caregiving (e.g. Solomon 2002; Wastel and White 2012; Koffman 2014). For instance, Vicedo (2011: 401– 402, 426) has argued that ‘Bowlby introduced a crucial new element’ into discourses on maternal responsibility with his ‘appeal to biology to justify the child’s need for mother anchored the old logic of functionalism upon a new scientific foundation, one that held a tremendous logical and emotional power.’ In particular, a substantial strand of Foucauldian scholarship has situated attachment theory as an important part of the discursive ‘software’ which operates the ‘hardware’ of the state’s biopolitical surveillance and policing of childrearing (e.g. Miller and Rose 1988; White 1996). These conclusions agree with the findings such as Holland (2001), whose study of child protection assessments in Britain found attachment used in the reasoning for the decision in every case; she also notes that attachment is the primary justification used when removing children from their families and placing them in care. Barth et al. (2005: 257) have described attachment as ‘the most popular theory for explaining parent–child behaviour by professionals and clinicians.’

We do not think that the significance of attachment can be reduced to its discursive construction and ideological deployment. Such a dismissal of attachment research can be illustrated with the case of Burman’s work. In *Deconstructing Developmental Psychology* (2007), she argues that attachment theory constructs distress in an incoherent way: ‘If the child will not settle to play some distance from her mother while she is there, the attachment is considered insecure. Conversely, this conclusion is also drawn if the child fails to protest at his or her mother’s departure’ (Burman 2007: 136). Burman suggests that attachment research is an ideological formation bent on pathologising working mothers with the incoherent claim that their children can only play if they are there and that the ‘greater independence’ a child might learn from going to day-care is a ‘measure of disturbance’ (Burman 2007: 136). However, the idea that infants show different degrees of distress depending on circumstances and their experiences is not incoherence but an insight. An infant who has a readily available caregiver is expected to be able to venture out in play; this is not by any means the primary criterion of security/insecurity, but provides some evidence towards making a coding. If a child is not able to use the caregiver as a safe base from which to explore, it suggests that the base does not feel so secure, and it is indeed the case that during the period of play with the caregiver, infants in relationships classified as avoidantly attached, in contrast to other infants, have very highly elevated cortisol levels (Luijk 2010). Conversely, an infant in a strange situation who is unexpectedly left by their caregiver is expected to be alarmed. Those who seem unbothered by their caregiver’s departure turn out, in fact, to have hidden signs of stress, such as a rapid heart rate. This suggests that they do feel alarmed when their caregiver leaves, but have learned that showing distress is counterproductive; home observations found that the caregivers of these infants tend to respond with rebuff when their infant is distressed and requests comfort (e.g. Isabella and Belsky 1991).

Attachment theory, then, is not reducible to an ideology bent on pathologising working mothers, rather it looks for clues to the texture of the wider encounter of child and caregiver in whether an infant uses their caregiver as a safe base from which to explore and communicates when they do feel distress. Attachment theory or research do not imply that day care is a problem. For instance, a study of over a thousand infants found that those who go to day care are only more likely to show insecure patterns of attachment in the strange situation when low caregiver sensitivity and responsiveness is combined with unpleasant day care (NICHD 1997). Or again, another study found that infants classified as securely attached in the strange situation were more likely to acclimatise quickly and effectively to day care and that some attachments changed from an insecure to a secure classification if the acclimatisation process was handled gently and sensitively (Anhert et al. 2004).

Yet, we do agree that attachment is also a discursive construction and that as such it has been deployed as a mandate for spurious claims and moral injunctions. As Berlant (1997) has documented, the figure of the infant is a particularly powerful one for neoliberal politics in policing the boundaries and formation of acceptable citizenship. An example is the significant role of attachment within contemporary British policy and political discourses, which has been debated in recent press coverage (e.g. Williams 2014; Meins 2014). In their influential report *Early Intervention: Good Parents, Great Kids, Better Citizens* Allen and Duncan Smith (2009) highlighted the importance of intervention to ensure that children’s attachment relationship with their mother is organised in a way that will produce obedient and self-sufficient citizens. They take two quotations from two attachment theorists to support these claims. From Shore (1994: 3), they extract the claim that ‘the child’s first relationship, the one with the mother, acts as a template that permanently moulds the individual’s capacity to enter into all later emotional relationships.’ They also present a quotation, which they attribute to Stern (1985): ‘because the infant’s cortical and hippocampal emotional circuits require significant time and experience to mature, the child must regulate its inner world primarily through attachment relationships’ (In fact, this passage is not to be found in Stern and has been lifted from Hosking and Walsh 2005: 19). Bringing the ideas of these two quotations together, Allen and Duncan Smith (2009) argued that the regulation of the infant’s emotional life within the infant’s attachment relationship with their mother must be regarded ‘as a prime requirement for a citizen to be of the law-abiding “self-regulator” type.’ The well-regulated infant will become the autonomous citizen of the neoliberal polis: ‘focusing on the first 3 years of children’s lives’ means ‘reducing dependence on the state’ among the citizens who will be produced (Allen and Duncan Smith 2009: 97); and this responsibility in turn will have a feed through to the next generation, who will receive more care and investment because their parents are able to have a ‘more responsible attitude to pregnancy and marriage’ (Allen and Duncan Smith 2009: 88).

Allen and Duncan Smith specify that whilst the middle classes can generally be expected and trusted to enact the regulated attachment relationship which will produce well-regulated citizens, this cannot be expected from others. The potential role of poverty, debt, housing problems, domestic violence or chronic health and mental illness in making caregiving more difficult is firmly bracketed by Allen and Duncan Smith (Grover and Mason 2013). Rather, like the Good Samaritan in the Gospel of Luke, the State must not ‘pass by on the other side’ but should sanction and regulate the mothers of potentially unregulated children—for the sake of the society which will otherwise have to suffer their behaviour:

Much of what we say here may not immediately appear relevant to middle class readers, whose children imbibe effective social behaviour unconsciously with their mother’s milk. However, even if conscience allowed us to pass by on the other side and say ‘nothing can be done’ our common sense would have to disagree, not least because the problems of the underclass or, as we call it here, the ‘dysfunctional base’, are leaching out into wider society (2009: 21).

Since 2010, Duncan Smith has served as Secretary of State for Work and Pensions, one of the most powerful figures in the Coalition Government. Duncan Smith has thus overseen both a growing focus on early attachment relationships within the Government’s Early Intervention agenda and the enactment of sweeping and controversial cuts to social security and welfare support to ‘return’ subjects to a state of individual responsibility (Gillies 2013). The intimacy between a focus on early familial relationships and a desire for the state to curb itself into a focus on support for markets appears at first sight mismatched. However, as Nadesan (2002) and Turner (2008) have observed, this is a necessary intimacy for the neoliberal polis, which requires of the family that it not only produces the kind of subjects adapted to be autonomous, responsible consumers and entrepreneurs, but also makes this process appear no more than the expression of a natural tendency. ‘Security’ is a powerful and polyvalent discourse (Hamilton 2013); since the Allen Review, it has had particular salience in British social policy, with attachment security framed as simultaneously the developmental scaffold for the socioeconomic security of the nation and as an argument against the need for social security and welfare supports provided by the State.

The significance of a child’s attachment for their development into autonomous and regulated citizens has received growing attention among policy makers. Speaking in the House of Commons, Annette Brooke, MP, has observed this shift: ‘I still have the original John Bowlby book, which was part of my set reading, on my bookshelf. It became a little unpopular because the women’s libbers of the 1970s were not too keen on the emphasis on mother staying at home, but it is interesting that attachment theory has come to the fore again’ (House of Commons Debate, 26 October 2010, c7WH). Andrea Leadsom, MP, highlights the significance of Main and Solomon’s concept of ‘disorganised attachment’ for this renewed interest in attachment:

A human being without a properly developed social brain finds it extremely difficult to empathise with other human beings. In particular, if a baby has what is known as disorganised attachment-where one or both parents are frightening or chaotic- they cannot form a secure bond precisely because the person who is so frightening and chaotic is also the person whom the baby should be turning to for comfort. The baby’s brain is confused and they experience disorganised attachment, which leads to very significant problems for that baby. If we look into the babyhood of children who brutalise other children, of violent criminals or of paedophiles, we can often see plenty of evidence that sociopaths are not born; rather they are made by their earliest experiences when they are less than 2 years old. Evidence shows that more than 80 % of long-term prison inmates have attachment problems that stem from babyhood. (House of Commons Debate, 26 October 2010, c1WH)

Leadsom has been firmly in favour of the Government’s welfare reforms, for example advocating for and voting to pass the Welfare Reform Act 2012 and the Welfare Benefits Up-rating Act 2013. For her, attachment is an issue unrelated to inequalities and welfare supports, since ‘poor attachment is no respecter of class or wealth’ (ibid.). This perspective permits her to filter out the potential causal significance of social and economic inequalities in shaping child outcomes, instead placing emphasis on the personal responsibility of parents for the personality of the child, which will then be set. A stark contrast is drawn between the affectability of the infant in their attachment relationship, and the causal autonomy of the personality thus shaped in setting a person’s capacity to be a law-abiding self-regulating citizen. Neoliberal policy lauds the minimal state, reduced to its sovereignty, penal and security services and succour for market forces; welfare support is framed as ‘interference with the laws of the market’ except where it is used to produce or support the creation of the markets or the citizen-consumer/entrepreneurs which are assumed to be both natural and optimal (Harcourt 2010; Davies 2014). Acknowledging that this would otherwise be her perspective, Leadsom proposes that using State institutions to screen families for disorganised attachment is different because disturbances of attachment undermine the conditions of adequate citizenship: ‘successive Governments have shied away from this proposition because intervention in the child/mother relationship can be intrusive. Indeed, the word “intervention” sounds just a little too much like interference. I would rather change the word, focusing in the opposite direction, and talk about prevention’ (House of Commons Debate, 26 October 2010, c1WH).

The same logic which pits attachment against welfare supports can also be seen in the recent Narey Report on Social Work Education (2014), which urged that social workers should be taught much more about ‘child development and attachment theory’ and much less about ‘non-oppressive practice’ (Narey 2014: 10). Narey observes that non-oppressive practice is a ‘thoroughly inadequate’ description of ‘the work of a Children’s Social Worker in England’: a social worker’s job is not to advocate for ‘principles of human rights and social justice’ but to deploy developmental theory in ‘protecting children’ (Narey 2014: 13). The Narey report offers a clear illustration of how attachment has been invoked to delegitimize social critique in the name of the needs of children and to contour the relationship between and respective responsibilities of the State and the family.

## Attachment and Subjection

As the example of recent political and policy discourses in Britain shows that attachment is partly a discursive construction—shaped by how we make meaning of it. However, we wish to draw out that attachment is not reducible to such constructions and as such may have characteristic properties and processes which are suggestive in themselves for sociological concerns. We turn now to Judith Butler, who situates attachment processes—not their construction—at the origin and as the mirror of the political formation of every subject. Considering Butler’s strong claims can help us to develop our position. Beginning from *The Psychic Life of Power* (1997), Butler repeatedly highlights the significance of the infant’s early attachment relationship as an object for social and political theory. ‘No subject emerges without a passionate attachment to those on whom he or she is fundamentally dependent’, she argues, and ‘this situation of primary dependency conditions the political formation and regulation of subjects and becomes the means of their subjection’ (Butler 1997: 7). This is a remarkable statement: the infant’s attachment is conceptualised as the irreducible paradigm for considering the political formation of subjects. Butler suggests that attachment is the missing affective explanation for why we submit to what Foucault calls disciplinary power, which assesses us against normative standards and causes us to regulate ourselves to achieve them. On the one hand, as Liberman et al. (2007: 179) have observed, this emphasis in Butler is indicative of ‘the far-ranging influence of attachment theory on contemporary thinking.’ On the other hand, Butler’s claim suggests the potential significance of attachment, not merely as an object of psychological knowledge among others, but as a necessary object for critical thought as the affective glue for disciplinary power.

The child’s attachment becomes the means of their subjection because the infant depends on the caregiver for protection and support, and so needs to seek them when alarmed; as such, the attachment system orients each human subject to emerge with feelings of love already present even for caregivers who are cruel or frightening. For Butler, the image of the infant attached to an abusive caregiver on the assumption that otherwise they can have no future serves, therefore, as both a condition and mirror of the adult political subject who emerges practically and affectively beholden to a normalising and potentially punitive culture:

Doubtless it seems better at that point to be enthralled with what is impoverished or abusive than not to be enthralled at all and so to lose the condition of one’s being and becoming. The bind of radically inadequate care consists of this, namely, that attachment is crucial to survival and that, when attachment takes place, it does so in relation to persons and institutional contexts that may well be violent, impoverishing, and inadequate. If an infant fails to attach, it is threatened with death, but, under some conditions, even if it does attach, it is threatened with non-survival from another direction… there are broader ethical consequences from this situation, ones that pertain not only to the adult world but to the sphere of politics and its implicit ethical dimension’ (1997: 45–6)

The ethical dimension of this predicament, Butler argues, is the same for each of us, no matter whether our early relationships were ‘loving and receptive’ or ‘a scene of abandonment or violence or starvation’: we emerge as subjects thrown into relations of love and need, into ways of understanding and practices which are not of our choosing; these establish the horizon for who we have become, before we are ever in a position to make further determinations (Butler 2004b: 24).

Butler specifies the scene of attachment as conditioning any and every process of subjection. This is a difficult, overly sweeping and perhaps painful thought. There is a sense of unease in commentators regarding whether Butler’s conceptualisation of attachment serves to welcome essentialism and political fatalism by the back door. It is not clear whether attachment phenomena are a biological, social or political processes for Butler (or how they might be all three) and what kind of mutability attachment might have under different conditions. For example, one of Butler’s strongest advocates, Mona Lloyd, states that the account of attachment is unusably flawed: ‘By positing the desire to exist as the substance that power exploits, Butler undercuts her own argument that psychic life is always already social. If the desire for existence is pre-discursive, then it is not only immune from criticism… it also cannot be changed by political intervention’ (Lloyd 2007: 102). Lloyd is concerned that when Butler suggests that attachment processes make us enthralled even to abusive caregivers and to punitive cultural forms, this implies that attachment is therefore prior to culture and as such cannot be changed by anything we do (see also McIvor 2012). To the extent that this is so, Allen and Duncan Smith would appear right to predict that well-regulated attachments in infancy would then seem aligned with the production of docile neoliberal subjects.

However, the charge that attachment is immune from opposition and cannot be resisted has been countered in Lauren Berlant’s essay ‘Love, a Queer Feeling’ (2001) and her later *Cruel Optimism* (2011). Unlike Butler, Berlant argues that not all subjection operates through attachment and calls for more attention to the boundaries and shape of attachment processes; she refers back to Bowlby and to empirical attachment research in considering the meanings and value of the concept of ‘attachment’—in amongst its usage as an ‘ideologeme’ (Berlant 2001: 449). What seems particularly to appeal to Berlant is less Bowlby’s own characterisation of attachment, than Ainsworth’s concept of attachment as the quality of one’s relationship with a secure base: she praises the term ‘attachment’, despite its flaws, as a ‘more neutral or impersonal’ way to address ‘an environment of touch or sound that you make so that there is something to which you turn and return’ (Berlant 2001: 439). Berlant agrees with Butler that the idea of attachment might well be the best way to engage with a vital question left behind by Foucault: why we emotionally invest in the cultures and institutions which discipline our identities and limit our potential to flourish (Berlant 2001: 441; see also Berlant 2012: 80). However, Berlant contests what she sees as a tendency in Butler to imply that ‘when adults imagine autonomy or sovereignty as synonymous with freedom, they are manifesting a humiliated reaction formation to having been duped, as an infant, into idealising a love that was always self-dispossessing and never not disappointing’ (Berlant 2011: 183).

While Butler’s emphasis is on survival and, correspondingly, on attachment as a scene of dependency, for Berlant the emphasis is on attachment as a scene through which children ‘organize their optimism for living … [making] do with what’s around that might respond adequately to their needs’ (Berlant 2011: 184). This reframing is subtle but crucial, since it points to features of the being of the child that exceed the immediate fact of dependency and the inevitability of subjection dependency implies for Butler. Conceived from the perspective of such a vital *conatus*, the scene of attachment appears less as the site for the negotiation of basic needs for protection with a concrete figure of power and more as the site where an affirmative impulse towards existence seeks recognition, acknowledgment, reciprocity and where ‘a noncoherent cluster of desires’ in this sense are able to converge into a ‘mirage of solidity’ through the figure of the object of love acting, we might say, as a focal lens Berlant (2011: 184). One theoretical advantage of this formulation is that it foregrounds the ontological ground on which the scene of attachment is predicated, rather than implying it by default as a consequence of taking dependency as a conceptual point of departure. Thus foregrounded, this ontological ground does not appear as a desire to exist that is destined, by the essential fact of the child’s dependency, to result in subjection to figures of power. On the contrary, in this reframing, the child’s desire to exist includes but is irreducible to the desire to survive, by virtue of the fact that the ‘child’ is defined by a relation to its multiple potentialities—by all that it could become—as much as its concrete (and dependent) actuality. The concept of an ‘optimism for living’ points to this dimension of potentiality whose possibilities, while finite, are radically indeterminate. In this sense, the desire to exist implicated in attachment relationships—*pace* Lloyd (2007)—points in a direction quite opposite to that of essentialist fatalism:

From this theoretical perspective on what love does to reproduce normativity, infantile dependency would not really be an experience of attaching to domination, but a scene where the subject negotiates an overdetermined set of promises and potentials for recognising and even thriving. It might be more like an environment where the subject is trained to cathect to optimism (Berlant 2011: 184).

Reflecting upon the attachments of her own early childhood, Berlant (2011: 125) relates: Mine was dominated by a general environment not of thriving but of disappointment, contempt, and threat. I salvaged my capacity to attach to persons by reconceiving of both their violence and their love as impersonal. This isn’t about me. This has had some unpleasant effects, as you might imagine. But it was also a way to protect my optimism… Out of this happy thought came an orientation towards fidelity to inclinations of all sorts, including those intellectual and political. Attachments are made not by will, after all, but by an intelligence after which we are always running (It’s not just “hey, you!” but “wait up!”).

Berlant does not specify at what age she began this process, which attachment theorists have conceptualised as the power of representational thought for reconstituting the self’s affective possibilities and patterns, where these have previously been subject to disorganising processes (Solomon et al. 1995; Baim & Morrison 2011). Attachment may begin before the cognitive infrastructure is sufficiently developed to fully engage such a strategy, but this does not stop the subject from yelling ‘hey, you’ (representing) and ‘wait up!’ (changing), whether these are attachments formed to threatening caregivers or to cultural forms. For Berlant, living out Butler’s scene of enthrallment to an impoverishing and violent caregiving environment is not an experience of early futility, to which the adult is simply the heir. Berlant documents that already as a child she was able to tactically reconceive the behaviours of her caregivers and in doing so alter her own possibilities for attachment, leaving room for optimism amidst impersonal forces and orienting by a fidelity to the texture of affect (which has become perhaps Berlant’s signature approach as a social theorist).

Indeed, Berlant describes how the optimism that she protected by refiguring her attachment serves as both the spur and object of her theoretical work and her political activism with the Feel Tank Chicago. This is possible because there is a disjuncture between attachment and subjectivity: The child is more than the product of their attachments, and their attachments offer a surprising excess of labile affect which disturb subjectivity and open other possibilities for becoming. The difference between a child and their attachment allows each of us to alter the terms of our enthrallment, representing and changing the terms established within our early relationships and subjection to cultural forms. As such, we do not have to fear or resent the power of these early imperatives. They do subject us to conditions which can be punitive and guide a tendency to accept and return to such conditions even later in life. However, this tendency can be countermanded as our attachment to particular figures or cultural forms is modified by the codetermination of subject and environment.

Yet, as well as elucidating the processes through which attachment and resistance can support one another, Berlant’s argument also develops Butler’s account of attachment by specifying how some attachments can be crueller than others. Whereas Butler is interested primarily in the fact that threatening or punitive caregiving does not release a child from their attachment, Berlant examines the affective economy which operates this process. Berlant proposes that ‘an optimistic attachment is cruel when the object/scene of desire is itself an obstacle to fulfilling the very wants that bring people to it: but its life-organising status can trump interfering with the damage it provokes’ (Berlant 2011: 227). A widespread and fundamental case of such attachment for adults, Berlant argues, is that ‘“the political” as we know it in mass democracy requires such a splitting of attachment and expectation. Splitting off political optimism from the way things are can sustain many kinds of the cruellest optimism’ (Berlant 2011: 228). Berlant thus urges recognition that if there is fatalism in the scene of attachment it lies not in a conceptualisation of the affects and relations that comprise attachment as immutable, but in the pervasiveness and enduring nature of social conditions in which cruel attachments to people, ideas or practices may be required as a means of psychical survival. Precisely, ‘what is cruel about these attachments, and not merely inconvenient or tragic, is that the subjects who have *x* in their lives might not well endure the loss of their object/scene of desire, even though its present threatens their well-being, because whatever the content of the attachment is, the continuity of its form provides something of the continuity of the subject’s sense of what it means to keep on living’ (Berlant 2011: 24).

## Sovereign Power

Butler highlights the significance of the scene and consequences of attachment; however, her account does not include an adequate account of attachment processes themselves and what about such processes mark it as political in binding and disturbing the formation of subjects. We need a sharper and less unilateral account of mechanisms. Helpful for such analysis is Berlant’s recognition of a disjuncture between the demands of attachment processes and the wider needs and conditions of flourishing for a human being. Many of Bowlby’s statements about the advantages for a child’s mental development of attentive mothering are premised on a conflation the two. As such, he tended to treat the availability of mother as the child’s primary need. He recognised but did not highlight that the reason a child wants their mother to be close is because attachment occurs initially with the most familiar adult, who in patriarchal societies tends to be the mother. He also recognised but did not highlight that making health, social and political resources available to a caregiver improves their capacity to offer sensitive care and the general emotional atmosphere of the home. Yet, following Butler and Berlant, we believe that there is value in analysing attachment for sociological significance—rather than either dismissing it as solely a social construction and ideology (à la Burman), or advocating for it as the will of Nature (à la Bowlby). Though we disagree with Bowlby’s political conclusions, his reflections on the integration of attachment as a motivational and behavioural system can themselves help us understand how attachment theory plugs in so smoothly to conservative discourses and to the operation of the psy disciplines.

Bowlby (1958: 369–370) compares the relationship of child to mother with that of citizens of the British Empire to their sovereign:

In healthy development it is towards her that each of the several responses becomes directed, much as each of the subjects of the realm comes to direct his loyalty towards the Queen; and it is in relation to the mother that the several responses become integrated into the complex behaviour which I have termed “attachment behaviour”, much as it is in relation to the Sovereign that the components of our constitution become integrated into a working whole.

We concur with critics such as Vicedo (2011) that such claims serve to justify and naturalise Bowlby’s conservative political and gender ideology through appeal to biology. However, we also wish to point out their acuity at a descriptive level for how attachment as a motivational and behavioural disposition seems to operate, and as to weigh and revise their theoretical significance. In his *Psychiatric Power* lecture series at the Collège de France, 1973–1974, Michel Foucault draws a distinction between sovereign and disciplinary schemas of power. These schemas represent, to Foucault, historically-specific apparatuses which have been significant, but never totalising, within Western society and institutions—though both have also been globalised in different ways (Valverde 2008). In Foucault’s account, sovereign power, particularly dominant in feudal Europe, has three defining characteristics. First, it enacts an intense individualisation of the person at the top of the hierarchy. Second, the relationship between sovereign and subject is given meaning by events in the past, ranging from right of birth to right of conquest. Third, Foucault follows Kantorowicz in arguing that the sovereign holds together in their person (as ‘the Crown’), and specifically in their body, the heterotopic relations under their jurisdiction, ranging from affective bonds to contractual obligations. The sovereign arbitrates and organises this entanglement of relations: ‘there is a need for something like a sovereign who, in his own body, is the point on which all these multiple, different and irreconcilable relationships converge’ (Foucault [1974] 2006: 45). In the legal framing of this relationship beginning from the thirteenth century, as Kantorowicz (1957: 374) documented, the sovereign is situated as the ultimate point of reconciliation and regulation for the nation because, specifically, the latter is a minor. By contrast, disciplinary power, characteristic of monastic orders and modern institutions such as barracks, schools and hospitals, is defined by its opposition as a schema of power on each of these points. First, disciplinary power is anonymous at the top, but enacts an intense individualisation at the bottom by situating each subject as responsible for their actions. Second, disciplinary power receives its legitimacy from its appeal to a ‘final or optimal state’ towards which it directs each subject ([1974] 2006: 42–47). Finally, Foucault suggests that it orchestrates possible relations of social and physical force in a progressive, graduated way; it rejects as pathological, and subjects to supplementary and more punitive disciplinary systems, those found to be residual to this discipline.

Foucault describes a general tendency for disciplinary power to spread across modern society. Yet, there is an anomaly: ‘it seems to me that the family is a sort of cell within which the power exercise is not, as one usually says, disciplinary, but rather of the same type as the power of sovereigns’ (Foucault [1974] 2006: 79). ‘In the family’, Foucault notes, ‘you have individualisation at the top’; ‘in the family there is constant reference to a type of bond, of commitment, and of dependence established once and for all in the form of marriage or birth.’ Lastly, ‘in the family there is all that entanglement of what could be called heterotopic relationships: an entanglement of local, contractual bonds, bonds of property, and of personal and collective commitments, which recalls the power of sovereignty rather than the monotony and isotopy of disciplinary systems’ (Foucault [1974] 2006: 80). This account permits Foucault to offer an explanation for the otherwise perplexing affective investment which is attached to otherwise grey disciplinary modes of subjection—Butler’s question, as we saw above. Foucault argues that the sovereign schema of power of the family, precisely through its difference, ‘is absolutely indispensible to the very functioning of all the disciplinary systems. I mean that the family is the instance of constraint that will permanently fix individuals to their disciplinary apparatuses… Look at how, historically, the obligation of military service was imposed on people who clearly had no reason to want to do their military service: it is solely because the State put pressure on the family’ (Foucault [1974] 2006: 81). Or again, ‘what meaning would the obligation to work have if individuals were not first of all held within the family’s system of sovereignty, within this system of commitments and obligations’ (Foucault [1974] 2006: 81). Foucault contends that the psy disciplines emerge precisely at the intersection between these two schema of power: ‘I think this really is the function of these psychologists, psychiatrists, criminologists, psychoanalysts, and the rest. What is their function if not to be agents of the organisation of a disciplinary power that will plug in, rush in, where an opening gapes in familial sovereignty?’ (Foucault [1974] 2006: 85).

We propose that Foucault’s analysis can be extended to argue that the attachment system parallels and supports this organisation of familial sovereignty, and its articulation with disciplinary power. Even in the developmental period before they discriminate themselves as an individuated subject, the behaviour shown by an alarmed infant to their attachment figure is itself centripetal and individualising. Whilst other caregivers will sometimes do and might achieve equal priority by the age of 2 months, during infancy the most familiar caregiver is generally preferentially discriminated as the object of attachment behaviour in the context of alarm. Unlike the law, which initially ties parent and child by virtue of the scene of birth, an infant is not more likely to attach to a birthparent compared to another equally available adult. However, the reference remains to the past rather than to the future since the attachment system directs an alarmed infant not to the most available or sensitive person in the present, but to the caregiver (and later, caregivers) with whom they have expectations of availability from the past (Umemura et al. 2013). Finally, the attachment system orients a centripetal relation to the body of a determinate caregiver (and later, caregivers); without such a relation, an infant would be unable to effectively regulate a heterotopic array of processes ranging from feelings and fantasies, to heart rate and hormone production (Hofer 2006; Cassidy et al. 2015). In sum, the demand for reunion of the infant alarmed by separation from their caregiver can be aligned with the schema of sovereign power, in which the corollary of the child’s enthrallment is the expectation of protection by the individualised caregiver. Since Aristotle, the sovereign has been conceptualised in the Western tradition as the source of political authority due to an attribution to the sovereign of self-sufficiency (*autarkeia*), of a status as an end in itself and hence a source of justification (*Politics*, 1253a 1–4). Just as subjects must orient themselves politically with reference to even an abusive sovereign due to the perception of the sovereign as the necessary ground, centre and responsible regulator of their political security in the face of potential external threats or the clash of internal forces (Agamben [2003] 2005; Loick 2012), so do the attachment behaviour of primate infants invest their caregiver with parallel expectations.

Genealogical research on attachment theory in the psy professions (Miller and Rose 1988; Vicedo 2011) has traced the role of popularisers of Bowlby’s ideas in spreading attachment theory to practitioners. To this research, we wish to add the proposal that each of the components of the schema of sovereign power in a child’s attachment behaviour help it to dovetail smoothly with the requirements of the psy disciplines in justifying individualising intervention. First, the intense individuation of the attachment figure, discriminated as the solution that the child demands for their distress, aligns with interventions which address the behaviour and personality of the parent with primary childcare responsibilities—often the mother—rather than the health, social and political resourcing of the caregiving system. In relation to the psy disciplines, interventions in which the resources available to caregivers are understood as incidental to their capacity to nurture children are generally preferred as cheaper and faster by statutory organisations with responsibility for health and social welfare (Krane et al. 2010). Such interventions also may have the utility of greater legitimacy in increasingly multicultural contexts, since assessments regarding health and harm can be measured against a universal psychological process shorn of social and political stakes. Second, the attachment system is oriented by the past rather than the present. This orientation is adapted for deployment as justification for psy disciplinary activity to optimise early caregiving experiences—in the context of the declining scientific credibility of psychoanalysis (cf. Donzelot [1977] 1997). Indeed, that attachment behaviour is observable and the attachment classifications have shown that substantial longitudinal significance in empirical studies has meant that deployment of attachment theory has been specifically been advocated by psy professionals as a response to the demand for evidence-based practice; Sheldon (2001: 803) describes how reference to Bowlby and Ainsworth among psy professionals has become a ‘Pavlovian reflex’ in justifying the scientific basis of their work with young children. Third and finally, the attachment system in infancy is organised around the body of the attachment figure, who is anticipated to regulate processes ranging from feelings to body temperature. Where the demands of the attachment system are conflated with the ultimate needs of the child and caregiver, this aligns with a perception of the family unit as naturally self sufficient or else pathological, and supports a conception of professional intervention as a matter of sufficient stakes to breach the privileged boundaries of the family and of individual freedom. To the degree that families are framed by policy makers and practitioners as occurring in a natural state of sovereign self-sufficiency, those families which deviate from this image do so as the result of irresponsible parenting rather than any lack of social and economic supports.

Conversely, where the demands of the attachment system are subject to scrutiny, other possibilities open up. As Berlant shows, attachment can enthral us to punitive situations and can precisely provide an opening onto change in our affective investments and circumstances. As applied to clinical and social welfare practice, consideration of attachment need not frame an image of familial sufficiency disrupted and then regained whilst individualising women as personally responsible rather than considering their access to economic, health, social or political resources. This image is a product of the lock between disciplinary and sovereign schemas of power, and a key point of weakness is that the sovereign schema finds its legitimacy in a reduction of the needs of the child to the demands of the attachment system. It is from the perspective of the infant’s attachment system that the familiar caregiver is like a self-sufficient sovereign: individualised, given meaning by past experience, and expected to manage incompatible feelings and relations. A different way of working with families is raised by both Berlant and attachment theory, which contests the image of sovereign power point by point. In this account, our attachments are a social achievement rather than a property of an individualised figure; they are open to the present and future rather than fixed by the past; and they never fully totalised the heterogeneity of our feelings and relations.

## Conclusion

As Berlant’s work demonstrates, the attachment-culture interface provides a key site of subjugation to the extent that our attachments bind us to ourselves, producing frames such as gender as a limit on who we can be, but it also offers a site of affectability and change. Beyond early childhood, new possibilities may arise or can be made which can help us respond creatively, mutually and politically when impelled by a tendency to seek safety in a milieu which does not support flourishing or does so at too high a price. To the extent that attachment remains a significant process within individual and cultural change, the call to find a safe base from which to explore does not need to be a clarion for a conservative focus on security as mere protection and individual responsibility but can serve as a spur to find bases for working for change together without expectations of harmony or wholeness, a more expansive security and/as exploration (Duschinsky et al. 2015).

One implication is that the politics of attachment change when the focus moves from a reified attachment system to the attachment-caregiving interface. From the beginning, the attachment system can only operate as a system because of its mesh with the caregiving system. The latter cannot be regarded as solely a personal property of the individualised caregiver but in turn is fully a social achievement, which can be clearly seen when it is not adequately resourced. Hrdy (2007) has documented that at a population level increasing the resources available to caregivers supports the nurturance they can offer to dependents and that decreasing such resources directly increases the likelihood that a caregiver will neglect their child. Attachment researchers have found that the individual caregiver’s sensitivity to their infant has less and less impact on that infant’s attachment the more the caregiver is deprived of economic, health, social and political resources (see De Wolff and van IJzendoorn 1997). The minimal State does not find justification in the child-caregiver relation; indeed, the findings and ideas of attachment theory can be helpful in demonstrating that discourses of familial or individual self-sufficiency are radically insufficient. Against those such as Allen and Duncan Smith who use a cartoon of attachment to support their insistence on a minimal role for the State in supporting its citizens, the findings of close attention to attachment processes—in Berlant’s social theory and empirical research with children alike—do not point away from but towards the importance for efforts to achieve social justice in order to provide the conditions for human flourishing.
